# Supplementary material for: Prevalence of HIV infection and related risk factors among young Thai men between 2010 and 2011
Source: PLoS One. 2020 Aug 14;15(8):e0237649. doi: 10.1371/journal.pone.0237649 (PMC7428352; doi:10.1371/journal.pone.0237649)
Supplement: S1 File — (PDF) [file pone.0237649.s001.pdf]

Risk factors for HIV infection among young Thai men (Questionnaires English version)

Instructions: Please read the questions carefully and answer them honestly by shading the circle that corresponds to your answers or place your answers on the space provided for.

I. Referencing
   1. ID No
   2. Date of filing

II. Demographic Profile
   1. What type of military personnel are you?
      a. Military Applicant
      b. Active Military Personnel
   2. Age (please indicate on the space provided)
      a. Your year of birth
      b. Your age (in years)
   3. What is your religion?
      a. Buddhism
      b. Christian
      c. Muslim
      d. Others (please specify on the space provided)
   4. Who are you living with?
      a. Parents
      b. Wife
      c. Relatives
      d. Friend
      e. Alone
      f. Others (Please specify on the space provided)
   5. Please indicate the province and district that you reside 2 years prior to today
      a. Province: ______________
      b. District: ________________
   6. Do you live in an urban or rural area
      a. Urban
      b. Rural
   7. What is your occupation
      a. Student
      b. Factory worker
      c. Laborer
      d. Unemployed
      e. Sales
      f. Fisherman/Farmer
      g. Mechant
      h. Others (please specify on the space provided): __________________
   8. What is your current civil status
      a. Married
      b. Divorced
      c. Widow
      d. Single
   9. What is your highest educational attainment
      a. No formal
      b. Primary education/ Elementary (PR 1-6)
      c. Middle school (Ma 1-3)
      d. High School (Ma 4-6)
      e. Vocational Highschool
      f. Vocational Certificate
      g. Vocational Diploma
      h. Bachelor's degree
      i. Others (please specify on the space provided): __________________

III. Risk Factors to HIV Infection

1. Have you ever used injectable drugs
   a. No/Never (if you answered No, please proceed to question #9)
   b. Yes (If you answered yes, please proceed to question# 2)
2. At what age did you first used injectable drugs (please indicate on the space provided): ________________
3. Since the beginning, what type of drug did you use (you can select more than one)
   a. Amphetamine
   b. Heroine
   c. Modicum
   d. Others (please specify on the space provided): __________________
4. In the last 12 months, what type of drug did you use (you can select more than one)
   a. Amphetamine
   b. Heroine
   c. Modicum
   d. Others (please specify on the space provided): __________________
5. Since the beginning, how often did you use this drug
   a. Only once
   b. Once a week
   c. Once a month
6. In the last 12 months, did you use new sterile needles to inject drugs to yourself?
   a. Yes
   b. No
7. Do you know where to get/buy new and sterile needle?
   a. No
   b. Yes (If yes, did you get from this place before?)
      1. Yes
      2. No
8. Have you ever been treated for drug addiction
   a. No/Never
   b. Yes (if yes, please indicate the rehabilitation center that you attended and location of facility)
9. In your lifetime, have you ever taken any non-injected addictive substances (aside from alcohol and cigarettes)
   a. No/Never (if no/never, please proceed to question#13
   b. Yes (If yes, please proceed to question #10)
10. In your lifetime, what type of non-injectable addictive drug have you used (you can choose more than 1)
    a. Amphetamine
    b. Heroine
    c. Opium
    d. Kratom
    e. Thinner/Solvent
    f. Sleeping Pills
    g. Party Drugs
    h. Combination Drugs
    i. Amphetamine
    j. Heroine
    k. Modicum
    l. Others (please specify on the space provided): __________________
11. Are you still using any of the drugs you have selected above
    a. Yes
    b. No
12. Have you ever been treated for drug addiction
    a. No/Never

  b. Yes (if yes, please indicate the rehabilitation center that you attended and location of facility)

13. Have you ever been in jail
    a. No/Never (if no, please proceed to question #18
    b. Yes
14. If yes, at what age were you in jail: _____
15. For how long were you in jail: _____
16. Where were you imprisoned
    a. Juvenile holding center
    b. Adult penitentiary
17. Please indicate the location of the jail that you were imprisoned on the space provided: ___________________________
18. Have you ever been tested for HIV?
    a. No/Never (if No/Never, please proceed to question #22)
    b. Yes
19. If you were tested before, what is the result
    a. Positive (if positive, please proceed to question #20)
    b. Negative (if negative, please proceed to question #21)
    c. I do not know (If you do now know, please proceed to question #21)
20. If you were tested positive, have you ever taken any drug for HIV infection
    a. Yes
    b. No

21. In the last 12 months, have you ever been tested for HIV
    a. Yes
    b. No
22. Do you know where to have yourself tested for HIV
    a. No
    b. Yes (If yes, please indicate the specific location): _______________
23. Are you circumcised?
    a. Yes
    b. No (if No, please answer question # 24)
24. If No, does the prepuce (head of the penis) free from skin or not
    a. Yes
    b. No
25. Have you ever had sex
    a. No/Never (if No/Never please proceed to question #)
    b. Yes
26. At what age have you had your first sexual experience
    a. Please indicate on the space provided: ___
27. With whom have you had your first sexual experience
    a. Girl friend
    b. Wife/common Law wife
    c. Female sex worker
    d. Female casual acquaintance
    e. Male casual acquaintance
    f. Male in exchange for gifts
    g. Transsexuals
    h. Others (please specify) ________
28. Have you ever had sex with a female sex worker
    a. Yes
    b. No
29. If yes, at what age did you first had sex with a female sex worker
    a. Please indicate on the space provided
30. How many sexual partners have you ever had
    a. Male sex partners

        1. Please indicate on the space provided
        2. I cannot remember/count
    b. Female sex partners
        1. Please indicate on the space provided
        2. I cannot remember/count

31. In the last 12 months, how many ***girlfriend/s*** have you had sex
    a. Please indicate in the space provided
    b. In the last 12 months, how often did you use condom while having sex with your ***girlfriend/s***
        1. Always
        2. Sometimes
        3. Never
    c. In the last sexual intercourse with your ***girlfriend/s***, did you use a condom
        1. Yes
        2. No
32. In the last 12 months, how many ***male lover/s*** have you had sex
    a. Please indicate in the space provided
    b. In the last 12 months, how often did you use condom while having sex with your ***male lover/s***
        1. Always
        2. Sometimes
        3. Never
    c. In the last sexual intercourse with your ***male lover/s***, did you use a condom
        1. Yes
        2. No
33. In the last 12 months, how many ***wife/ves*** have you had sex
    a. Please indicate in the space provided
    b. In the last 12 months, how often did you use condom while having sex with your ***wife/ves***
        1. Always
        2. Sometimes
        3. Never
    c. In the last sexual intercourse with your ***wife/ves***, did you use a condom
        1. Yes
        2. No
34. In the last 12 months, how many ***female sex workers/s*** have you had sex
    a. Please indicate in the space provided
    b. In the last 12 months, how often did you use condom while having sex with a ***female sex worker/s***
        1. Always
        2. Sometimes
        3. Never
    c. In the last sexual intercourse with a ***female sex worker/s***, did you use a condom
        1. Yes
        2. No
35. In the last 12 months, how many ***male sex worker/s*** have you had sex
    a. Please indicate in the space provided
    b. In the last 12 months, how often did you use condom while having sex with your ***male sex worker/s***
        1. Always
        2. Sometimes
        3. Never
    c. In the last sexual intercourse with your ***male sex worker/s***, did you use a condom
        1. Yes
        2. No
36. In the last 12 months, how many ***casual male acquaintance/s*** have you had sex

   a. Please indicate in the space provided
   b. In the last 12 months, how often did you use condom while having sex with a ***casual male acquaintance/s***
      1. Always
      2. Sometimes
      3. Never
   c. In the last sexual intercourse with a ***casual male acquaintance***, did you use a condom
      1. Yes
      2. No

37. In the last 12 months, how many ***casual female acquaintance/s*** have you had sex
   a. Please indicate in the space provided
   b. In the last 12 months, how often did you use condom while having sex with a ***casual female acquaintance/s***
      1. Always
      2. Sometimes
      3. Never
   c. In the last sexual intercourse with a ***casual female acquaintance***, did you use a condom
      1. Yes
      2. No
38. In the last 12 months, how many ***male partners other than the specified above*** have you had sex
   a. Please indicate in the space provided
   b. Please indicate your relationship with him
   c. In the last 12 months, how often did you use condom while having sex with a ***other male acquaintance/s***
      1. Always
      2. Sometimes
      3. Never
   d. In the last sexual intercourse with a ***other male acquaintance***, did you use a condom
      1. Yes
      2. No
39. In the last 12 months, how many ***female partners other than the specified above*** have you had sex
   a. Please indicate in the space provided
   b. Please indicate your relationship with him
   c. In the last 12 months, how often did you use condom while having sex with a ***other female acquaintance/s***
      1. Always
      2. Sometimes
      3. Never
   d. In the last sexual intercourse with a ***other female acquaintance***, did you use a condom
      1. Yes
      2. No
40. Have you ever had sex with another man?
   a. No/Never (If no/never, please proceed to question #)
   b. Yes
41. If yes, have you ever had anal sex with another man
   a. Never
   b. Yes
42. If you ever had anal sex with another man, what is your relative position
   a. Receptive
   b. Insertive

   c. Both
43. If both, which is predominant in terms of frequency
   a. Receptive
   b. Insertive
   c. Equal in frequency
44. During ***Insertive anal sex*** with another man, did you use condom
   a. Yes
   b. No
45. During ***Receptive anal sex*** with another man, did you use condom
   a. Yes
   b. No
46. How did you meet the man that you had sex with
   a. Internet
   b. Introduced with a friend
   c. Introduced by a relative
   d. Cinema
   e. Park
   f. Fitness place
   g. Anywhere
   h. Entertainment place
      1. Pub/Bar
      2. Gay Bar
      3. Disco
      4. Small stall
      5. Brothel
      6. Karaoke
      7. Restaurant
      8. Sauna
      9. Others, (please specify: _____________)
   i. Others, (Please specify: _____________)
47. Where did you had sex
   a. Dormitory
   b. House
   c. Hotel
   d. Cinema
   e. Sauna
   f. Pop bar
   g. Spa/massage
   h. Public toilet
   i. Farm cottage
   j. Park
48. What province did you had sex with another man
   a. Bangkok
   b. Outside Bangkok
49. Which part of the province did you had sex with another man
   a. Urban
   b. Rural
50. In the last 12 months, Have you ever had any sexually transmitted infection
   a. No/Never
   b. Yes
51. If yes, what type of infection (you can choose more than one)
   a. Syphilis
   b. Gonorrhea
   c. Chlamydia
   d. Herpes
   e. LGU

   f. Chondyloma
   g. Problem urination
   h. Local sexual lesion
   i. Nodules
   j. Pimple
   k. Others (please specify: _________

52. How did you treat the sexually transmitted infection
    a. Personal
    b. Clinic
       1. Government
       2. Private
    c. Hospital
       1. Government
       2. Private
    d. Did nothing
    e. Others, please specify: ________________
53. Have you ever had sex in exchange for gifts
    a. No/Never
    b. Yes
54. If you ever had sex with another man in exchange for gifts, how many times, please specify: _____________
55. If you ever had sex with another in exchange for gifts, what is your relationship with the man
56. Have you ever had been forced to have sex
    a. No
    b. Yes
57. What is the gender of the person who forced you to have sex
    a. Male
    b. Female
58. What is your gender preference
    a. Male
    b. Female
    c. Both male and female
59. Do you know where to get condom
    a. No
    b. Yes
60. Where do you get condom
    a. Small health center
    b. Hospital
    c. Others, please specify: _________________
61. Do you know where to buy condom:
    a. No
    b. Yes
62. Where do you buy condom
    a. Convenient store
    b. Drug store
    c. Automatic machine
    d. Others (please specify: ________________)
63. Knowledge Questions: Please answer the following questions based on your knowledge about HIV infection
    a. Do you know that the use of condom can prevent HIV/AIDS transmission
       1. Yes
       2. No
    b. Do you know, that having only one sexual partner who have no history of HIV/AIDS can prevent HIV/AIDS transmission
       1. Yes

2. No

c. HIV/AIDS can be transmitted by mosquito bites
    1. Yes
    2. No
d. HIV/AIDS can be transmitted by sharing food
    1. Yes
    2. No
e. A person who look healthy can be infected with HIV
    1. Yes
    2. No
f. Now a days, there are drugs against HIV (antiretrovirals)
    1. Yes
    2. No
g. HIV can be transmitted by intravenous drug use
    1. Yes
    2. No
