# Supplementary material for: Prevalence of HIV infection and related risk factors among young Thai men between 2010 and 2011
Source: PLoS One. 2020 Aug 14;15(8):e0237649. doi: 10.1371/journal.pone.0237649 (PMC7428352; doi:10.1371/journal.pone.0237649)
Supplement: S2 File — (PDF) [file pone.0237649.s002.pdf]

Risk factors for HIV infection among young Thai men (Questionnaires Thai version)

 Data Dict รหัสแบบสอบถาม ID

แบบสอบถามสำหรับทหารกองประจำการ

(คำตอบของท่านจะถูกเก็บเป็นความลับอย่างเคร่งครัด ท่านไม่ต้องระบุชื่อ และขอได้โปรดตอบตามความเป็นจริง)

คำชี้แจง กรุณาทำเครื่องหมาย X ในช่อง ○(ตัวอย่าง ⊗)หรือเติมคำใน.......... หรือตัวเลขในช่องว่าง ☐

ในกรณีที่เลือกผิด ให้ขีดทับ 2 เส้น (ตัวอย่าง ⊗) แล้วเลือกข้อใหม่

หากต้องการเลือกข้อเดิม ให้ใส่คำว่า"เอา"บนขวามือบนข้อที่เลือก (ตัวอย่าง ⊗ เอา)

57 | 601

วันที่ทำแบบสอบถาม............/...date.../........................(วัน/เดือน/ปี พ.ศ. ตัวอย่าง 01 /ม.ค. /2553)

1. ท่านเข้าเป็นทหารเกณฑ์แบบใด → military ○ 1.สมัครเข้าเป็นทหาร ○ 2.ผ่านการเกณฑ์ทหาร

2. ท่านเกิดปี พ.ศ. bth_yr ☐☐☐☐ (ตัวอย่างพ.ศ. 2527) ปัจจุบันท่านอายุเท่าไร age ☐☐ ปี (ตัวอย่าง 20 ปี)

3. ท่านนับถือศาสนาใด → relig ○ 1. พุทธ ○ 2. คริสต์ ○ 3. อิสลาม ○4. อื่นๆ ระบุ.........relig..............

4. ก่อนมาเป็นทหารท่านพักอาศัยกับใคร

stay ○ 1. พ่อแม่ ○ 2.แฟน/ภรรยา ○ 3.ญาติ ○ 4. เพื่อน ○ 5.อยู่คนเดียว ○ 6.อื่นๆ ระบุ ...stayX..

5. ภูมิลำเนาในระยะเวลา 2 ปี ก่อนเข้าเป็นทหารท่านอยู่ที่ใดนานที่สุด

จังหวัด (ระบุ)..........prov..........อำเภอ (ระบุอำเภอเดียว)..........dist.......... และอยู่ในเขตเทศบาลหรือนอกเขตเทศบาล

town ○ 1.ในเขตเทศบาล/สุขาภิบาล ○ 2.นอกเขตเทศบาล/สุขาภิบาล

6. ก่อนมาเป็นทหารท่านประกอบอาชีพอะไรเป็นอาชีพหลัก

occ
○ 1.นักเรียน/นักศึกษา ○ 2.ลูกจ้างโรงงาน/ บริษัท ○ 3.กรรมกร/ผู้ใช้แรงงาน
○ 4.ว่างงาน ○ 5.ลูกจ้างร้านค้า ○ 6.ประมง/ทำนา/ทำไร่
○ 7.ค้าขาย ○ 8.อื่นๆ ระบุ ....occX............

7. ปัจจุบันนี้สถานภาพสมรสของท่านคือ → status ○ 1.แต่งงาน/อยู่ด้วยกันเฉยๆ ○ 2.หย่า/แยกกันอยู่ ○ 3.หม้าย ○ 4.โสด

8. ท่านเรียนหนังสือสูงสุดถึงชั้นใด

edu
○ 1. ไม่ได้เรียน ○ 2. ป.1 - ป.6 ○ 3. ม.1 - ม.3 ○ 4. ม.4 - ม.6 ○ 5. ปวช.
○ 6. ปวส. ○ 7. อนุปริญญา ○ 8. ปริญญาตรี ○ 9. อื่นๆ..........eduX..........

9. ก่อนมาเป็นทหาร ท่านเคยใช้สารเสพติดโดย**การฉีดเข้าเส้นเลือด**หรือไม่

inj
○ 1. ไม่เคย **ข้ามไปตอบข้อที่ 11**
○ 2. เคย**ถ้าเคย ให้ตอบ ข้อที่ 9.1 - 9.4** ท่านเริ่มใช้ครั้งแรกเมื่ออายุ inj_age ☐☐ ปี

9.1 สารเสพติดชนิดใดบ้างที่ท่านเคยใช้ฉีดเข้าเส้นเลือด (ตอบได้มากกว่า 1 ข้อ)

Inj1 ○ 1.ยาบ้า Inj2 ○ 2.เฮโรอีน Inj3 ○ 3.ดอมิกุม (ยาเม็ด) Inj4 ○ 4.อื่นๆ ระบุ ....Inj4X..........

9.2 **ในช่วง 12 เดือนที่ผ่านมา** ท่านยังใช้สารเสพติดโดยการฉีดเข้าเส้นเลือดอยู่หรือไม่

Inj_y
○ 1.ไม่ได้ใช้แล้ว **(ข้ามไปตอบข้อที่ 9.3)**
○ 2.ยังใช้อยู่ **ถ้ายังใช้อยู่** สารเสพติดชนิดใดบ้างที่ท่านยังใช้ฉีดเข้าเส้นเลือด

Inj_yu1 ○ 1.ยาบ้า Inj_yu2 ○ 2.เฮโรอีน Inj_yu3 ○ 3.ดอมิกุม (ยาเม็ด) Inj_yu4 ○ 4.อื่นๆ ระบุ..........Inj_yu4X..........

และ ท่านใช้บ่อยแค่ไหน → Inj_yuf ○ 1.เพียงครั้งเดียว ○ 2. 1 ครั้ง/สัปดาห์ ○ 3. 1 ครั้ง/เดือน

9.3 **ในช่วง 12 เดือนที่ผ่านมา** ท่านใช้เข็มฉีดยาใหม่ในการฉีดยาเสพติดครั้งล่าสุดหรือไม่

Inj_syry ○ 1.ใช้ ○ 2.ไม่ได้ใช้

9.4 ท่านทราบหรือไม่ว่า สามารถรับแจกเข็ม/ กระบอกฉีดยาใหม่ ได้จากที่ใด

Inj_syrplc ○ 1.ไม่ทราบ ○ 2.ทราบ **ถ้าทราบ** ท่านเคยไปรับบริการหรือไม่ → Inj_syrplcX ○ 1.เคย ○ 2.ไม่เคย

รหัสแบบสอบถาม ______ ID

10. **จากคำถามข้อที่ 9** ท่านเคย**ได้รับการรักษา/ บำบัด** การติดยาเสพติดชนิด**ฉีดเข้าเส้นเลือด** หรือไม่

inj_tr
○ ไม่เคย
○ เคย **ถ้าเคย** โปรดระบุสถานที่ที่ท่านได้รับการรักษา .... inj_trX ....

11. ก่อนมาเป็นทหาร ท่านเคยใช้สารเสพติดโดย**การกิน สูบ หรือสูดดม**หรือไม่ (ไม่นับบุหรี่ เหล้าหรือเบียร์)

whiff
○ 1. ไม่เคย **ข้ามไปตอบข้อที่ 12**
○ 2. เคย**ถ้าเคยให้ตอบ ข้อที่ 11.1 - 11.3**

11.1 สารเสพติดชนิดใดบ้างที่ท่านเคยใช้ (ตอบได้มากกว่า 1 ข้อ)

Whiff1 ○ 1.ยาบ้า Whiff2 ○ 2.ใบกระท่อม Whiff3 ○ 3.ยาเค/ยาอี/ยาเลิฟ/ยาไอซ์ Whiff4 ○ 4.เฮโรอีน
Whiff5 ○ 5.ทินเนอร์/กาว Whiff6 ○ 6.กัญชา/ฝิ่น Whiff7 ○ 7.ยานอนหลับ(ดอมิกุม)
Whiff8 ○ 8.สี่คูณร้อย/เชือน/แพนาดิ้ว/วันทูคอล Whiff9 ○ 9.อื่นๆ........ Whiff9X ....

11.2 ปัจจุบันท่านยังใช้สารเสพติดโดย การกิน สูบ หรือ สูดดม อยู่หรือไม่

Whiff_u ○ 1.ยังใช้อยู่ ○ 2.ไม่ได้ใช้แล้ว

11.3 ท่านเคย**ได้รับการรักษา**อาการติดยาเสพติดชนิด**กิน สูบ หรือสูดดม** หรือไม่

Whiff_tr
○ ไม่เคย
○ เคย **ถ้าเคย** โปรดระบุสถานที่ที่ท่านได้รับการรักษา .... Whiff_trX ....

12. ก่อนมารับราชการทหาร **ท่านเคยต้องโทษ ถูกจำคุก หรือถูกคุมขัง** หรือไม่

jail
○ 1. ไม่เคย **ข้ามไปตอบข้อที่ 13**
○ 2. เคย **ถ้าเคย** จังหวัดใด........ Jail_prov .... เมื่ออายุ □□ (Jail_age) ปี เป็นระยะเวลา □□ (Jail_yr) ปี □□ (Jail_m) เดือน

และท่านเคยต้องโทษหรือถูกจำคุกที่ใด Jail_plc1 ○ 1. สถานพินิจฯ Jail_plc2 ○ 2. เรือนจำ
Jail_plc3 ○ 3. อื่น ๆ ระบุ........ Jail_plc3X ....

13. ก่อนมารับราชการทหาร ท่านเคยได้รับการตรวจเลือดหาเชื้อ เอชไอวี หรือเอดส์ บ้างหรือไม่

chk_hiv ○ 1.เคยได้รับการตรวจ **ถ้าเคย** ผลการตรวจของท่านคือข้อใด

chk_dis
○ 1.เป็นโรค **ถ้าเป็นโรค** ท่านได้รับยาต้านเชื้อไวรัสหรือไม่ → chk_drug ○ 1.ได้รับ ○ 2.ไม่ได้รับ
○ 2.ไม่เป็นโรค
○ 3.ไม่ทราบผล

และ ในช่วง 12 เดือนที่ผ่านมา เคยได้รับการตรวจหาเชื้อ เอชไอวี หรือไม่ → chk_yr ○ 1. ตรวจ ○ 2. ไม่ได้ตรวจ

○ 2.ไม่เคยได้ตรวจเลย **ถ้าไม่เคย** ท่านทราบหรือไม่ว่าสามารถตรวจเลือดเพื่อหาการติดเชื้อเอชไอวีได้จากที่ใด

chk_plc ○ 1. ไม่ทราบ ○ 2. ทราบ ระบุสถานที่........ chk_plcX ....

14. ท่านเคยได้**รับเลือด**จากโรงพยาบาล เนื่องจากอุบัติเหตุ/ โรคเลือดบางชนิด/ การเจ็บป่วยอื่น ๆ หรือไม่

bld_rec ○ 1. เคย ○ 2. ไม่เคย

15. ท่าน **เคยขริบ** หนังหุ้มปลายอวัยวะเพศ หรือไม่

cut
○ 1.เคย
○ 2.ไม่เคย **ถ้าไม่เคยขริบ** หนังหุ้มปลายอวัยวะเพศ ของท่านเปิดหรือยัง → Cut_open ○ 1.เปิดแล้ว ○ 2.ยังไม่เปิด

 รหัสแบบสอบถาม ID

16. ท่านเคยร่วมเพศหรือไม่ **นับรวมไม่ว่าจะกับผู้หญิงหรือผู้ชาย**ก็ตาม

sxfst
- ○ 1.ไม่เคยร่วมเพศ **ข้ามไปตอบข้อที่ 26**
- ○ 2.เคยร่วมเพศ ท่านร่วมเพศครั้งแรกเมื่ออายุ ☐☐ ปี(ตัวอย่าง 2 5 ปี) Sxfst_age

17. ท่านร่วมเพศครั้งแรกกับใคร ไม่ว่าจะเป็นหญิงหรือชาย

Sxfst_who
- ○ 1.เพื่อนหญิง/ คนรัก/เพื่อนสนิท
- ○ 2.ภรรยา/ ผู้หญิงที่อยู่กินด้วยกันในปัจจุบัน
- ○ 3.หญิงขายบริการทางเพศ/โสเภณี
- ○ 4.ผู้หญิงอื่น (นอกจากข้อ 1, 2, 3)
- ○ 5.ผู้ชายทั่วไปไม่นับรวมผู้ชายที่ขายบริการทางเพศ
- ○ 6.ผู้ชายขายบริการทางเพศ
- ○ 7.กะเทย/ กะเทยขายบริการทางเพศ
- ○ 8.อื่นๆ ระบุ.........Sxfst_whoX.........

18. ท่านเคยร่วมเพศกับ**หญิงขายบริการทางเพศ**หรือไม่

sxsvfm
- ○ 1.เคยท่านร่วมเพศกับหญิงขายบริการทางเพศครั้งแรกเมื่ออายุ ☐☐ ปี(ตัวอย่าง 2 5 ปี) Sxsvfm_age
- ○ 2.ไม่เคยร่วมเพศกับหญิงขายบริการทางเพศ

19. **ตลอดชีวิตที่ผ่านมา** คนที่ท่านร่วมเพศด้วยมีทั้งหมด จำนวน ☐☐ คน (sxsum) ○ นับไม่ถ้วน (sxsumX)

แบ่งเป็นเพศหญิงหรือเพศชายจำนวนกี่คน
- → เป็น เพศหญิง จำนวน ☐☐ คน (sxsumfm) ○นับไม่ถ้วน (sxsumfmX)
- → เป็น เพศชายรวมกะเทย จำนวน ☐☐ คน (sxsumm) ○นับไม่ถ้วน (sxsummX)

20. **ในช่วง 12 เดือนที่ผ่านมา** คนที่ท่านร่วมเพศด้วยมีใครบ้าง เป็นเพศใด และมีจำนวนเท่าไหร่ (เลือกได้หลายคำตอบ)
กรุณาทำเครื่องหมาย X ในช่อง○(ตัวอย่าง ⊗)ที่ตรงกับความเป็นจริง (* ชาย หมายถึง เพศชาย รวมทั้งกะเทยด้วย)

ประเภทบุคคล	เพศ	จำนวน ตัวอย่าง 2 5 คน	การใช้ถุงยางอนามัย ในช่วง 12 เดือนที่ผ่านมา นับรวมคู่นอนทุกคู่			ร่วมเพศครั้งล่าสุด ใช้ถุงยางอนามัยหรือไม่	
			ใช้ทุกครั้ง	ใช้บางครั้ง	ไม่ได้ใช้	ใช้	ไม่ใช้
คนรัก/ เพื่อนสนิท/ คู่นอนประจำ/ กิ๊ก	ชาย*	sxlvm คน⇨	○ sxlvmcon	○	○	○ sxlvmclst	○
	หญิง	sxlvfm คน⇨	○ sxlvfmcon	○	○	○ sxlvfmclst	○
ภรรยา/เมีย (ทั้งจดและไม่จดทะเบียน)	หญิง	sxmrfm คน⇨	○ sxmrfmcon	○	○	○ sxmrfmclst	○
ผู้ขายบริการทางเพศ/ คู่นอนที่ต้องจ่ายเงิน	ชาย*	sxsvm คน⇨	○ sxsvmcon	○	○	○ sxsvmclst	○
	หญิง	sxsvfm คน⇨	○ sxsvfmcon	○	○	○ sxsvfmclst	○
คนที่เพิ่งรู้จัก/ มีความสัมพันธ์ชั่วคราว	ชาย*	sxmtm คน⇨	○ sxmtmcon	○	○	○ sxmtmclst	○
	หญิง	sxmtfm คน⇨	○ sxmtfmcon	○	○	○ sxmtfmclst	○
อื่นๆ.............. sxotX	ชาย*	sxotm คน⇨	○ sxotmcon	○	○	○ sxotmclst	○
	หญิง	sxotfm คน⇨	○ sxotfmcon	○	○	○ sxotfmclst	○

 รหัสแบบสอบถาม ______ ID

Anal

21. ท่านเคยมีความสัมพันธ์ทางเพศกับผู้ชายด้วยกัน หรือไม่

msm ○ 1.ไม่เคย **ข้ามไปตอบข้อที่ 24**

○ 2.เคย **ถ้าเคย**ท่านร่วมเพศแบบมีการสอดใส่ทางทวารหนักหรือไม่ ⟶ Msm_in ○1.ไม่เคย ○2. เคย

ถ้าเคย ในการร่วมเพศกับผู้ชายท่านเป็นฝ่ายใด

Msm_2fb ○ 1.เป็นทั้งฝ่ายรุก**(ผู้สอดใส่)**และฝ่ายรับ**(ผู้ถูกสอดใส่)**โดย**มีอย่างใดมากกว่ากัน**

Msm_fwbw ○ 1.รุกมากกว่ารับ ○ 2.รับมากกว่ารุก ○ 3.รุกรับพอๆกัน

○ 2.ฝ่ายรุกอย่างเดียว**(ผู้สอดใส่)**ถ้าท่านเป็นฝ่ายรุกในการร่วมเพศครั้งล่าสุด

ท่านสวมถุงยางอนามัยหรือไม่ ⟶ Msm_fw ○ 1.สวม ○ 2. ไม่สวม

○ 3.ฝ่ายรับอย่างเดียว **(ผู้ถูกสอดใส่)** ถ้าท่านเป็นฝ่ายรับในการร่วมเพศครั้งล่าสุด

ผู้ที่เป็น**ฝ่ายรุกสวมถุงยางอนามัยหรือไม่** ⟶ Msm_bw ○ 1.สวม ○ 2. ไม่สวม

22. ท่าน **รู้จัก / พบ / เจอ** ผู้ชายด้วยกันที่เป็นคู่นอนของท่านได้อย่างไร (ตอบได้มากกว่า 1 ข้อ)

Msm1 ○ 1. อินเตอร์เน็ต (internet) Msm2 ○ 2. เพื่อนแนะนำ (Friend) Msm3 ○ 3.ญาติในครอบครัว (Family/Relative) Msm4 ○ 4. โรงภาพยนตร์ (Theaters)

Msm5 ○ 5. สวนสาธารณะ (park) Msm6 ○ 6. ฟิตเน็ตเซ็นเตอร์ (Fitness Center) Msm7 ○ 7. ซื้อบริการทางเพศไม่ว่าจากแหล่งใดก็ตาม**ที่ต้องจ่ายเงิน** (Com)

Msm8 ○ 8. สถานบันเทิง (Entertaiment venues) **โปรดเลือกประเภทของสถานบันเทิง** (ตอบได้มากกว่า 1 ข้อ)

Msm81 ○ 8.1 ผับ/บาร์ Msm82 ○ 8.2 บาร์เกย์ Msm83 ○ 8.3 ดิสโก้เทค Msm84 ○ 8.4 ร้านเหล้า/เหล้าปั่น

Msm85 ○ 8.5 อาบอบนวด/สปา Msm86 ○ 8.6 ร้านคาราโอเกะ Msm87 ○ 8.7 ร้านอาหาร Msm88 ○ 8.8 ซาวน่า

Msm89 ○ 8.9 อื่นๆ ระบุ .... Msm89X ....

Msm9 ○ 9. อื่นๆ ระบุ .... Msm9X ....

เหมือน 1 ตัวแปร.

23. **ครั้งล่าสุด**ที่ท่านมีเพศสัมพันธ์ **กับคู่นอนที่เป็นผู้ชายด้วยกัน**ของท่านในสถานที่ใด (ตอบได้ข้อเดียว)

Msmplc1 ○ 1. หอพัก/ห้องเช่า Msmplc2 ○ 2. บ้านพัก Msmplc3 ○ 3. โรงแรม

Msmplc4 ○ 4. โรงภาพยนตร์ Msmplc5 ○ 5. ซาวน่า Msmplc6 ○ 6. ผับ/บาร์/สถานบันเทิง ⇨

Msmplc7 ○ 7. ร้านนวด/สปา Msmplc8 ○ 8. ห้องน้ำตามสถานที่สาธารณะ

Msmplc9 ○ 9. เถียงนา/ไร่นา Msmplc10 ○ 10. สวนสาธารณะ

Msmplc11 ○ 11.อื่น ๆ ระบุ.... Msmplc11X ....

สถานที่ที่ท่านมีเพศสัมพันธ์

Msmprov ○ 1. กรุงเทพมหานคร

○ 2. ต่างจังหวัด

Msmprovt ○ 2.1 ในเขตเทศบาล

○ 2.2 นอกเขตเทศบาล

24. **ในช่วง 12 เดือนที่ผ่านมา** ท่านมีอาการ หรือเคยได้รับการตรวจว่าเป็นโรคติดต่อทางเพศสัมพันธ์ บ้างหรือไม่

sxdis_y ○ 1. ไม่เคย **ข้ามไปตอบข้อที่ 25**

○ 2. เคย **กรณีเคย**โปรดระบุโรคที่ท่านเป็น (ตอบได้มากกว่า 1 ข้อ)

Sxdis1 ○ 1. ซิฟิลิส Sxdis2 ○ 2. หนองในแท้ Sxdis3 ○ 3. หนองในเทียม Sxdis4 ○ 4. เริม

Sxdis5 ○ 5. ไข่ดันบวม/ ฝีมะม่วง Sxdis6 ○ 6. หูดหงอนไก่บริเวณอวัยวะเพศ Sxdis7 ○ 7. ปัสสาวะบ่อยและแสบขัด

Sxdis8 ○ 8. แผลลักษณะอื่นๆบริเวณอวัยวะเพศ Sxdis9 ○9. มีผื่น/ ตุ่มน้ำที่อวัยวะเพศหรือทวารหนัก

Sxdis10 ○ 10. มีหนอง/ น้ำใสๆ/น้ำเหลือง ซึมออกจากปลายอวัยวะเพศ Sxdis11 ○ 11. อื่นๆ ระบุ .... Sxdis11X ....

และ โดยส่วนใหญ่ท่านรักษาอย่างไร

sxdistr ○ 1. ซื้อยากินเอง

○ 2. ไปคลินิก, ศูนย์กามโรค อยู่ในสังกัดของ ⟶ sxdistr_2g ○ 1. รัฐ ○ 2. เอกชน

○ 3. โรงพยาบาล อยู่ในสังกัดของ ⟶ sxdistr_3g ○ 1. รัฐ ○ 2. เอกชน

○ 4. ไม่ทำอะไรเลย

○ 5. อื่นๆ ระบุ .... sxdistrX ....

 รหัสแบบสอบถาม ______ ID ______

25. ท่านเคยมีเพศสัมพันธ์โดยได้รับ<u>เงิน/ สิ่งของ</u> หรือไม่

sx_mn
- ○ 1. ไม่เคย **(ข้ามไปตอบข้อที่ 26)**
- ○ 2. เคย

sx_mnf

<u>**ถ้าเคย ในช่วงเวลา 12 เดือนที่ผ่านมา**</u>ท่านเคยมีเพศสัมพันธ์โดยได้รับ เงิน/ สิ่งของ <u>**กี่ครั้ง**</u> ☐☐ ครั้ง <u>**และ ท่านมีเพศสัมพันธ์**</u>โดยได้รับเงิน/ สิ่งของ จากใครบ้าง (เช่น คนต่างชาติ, เพื่อน, อาชีพอะไร เป็นต้น) โปรดระบุ.................. sx_mnfX ....

26. ท่านเคยถูกบังคับขืนใจให้มีเพศสัมพันธ์หรือไม่

sx_rape
- ○ 1. ไม่เคย
- ○ 2. เคย ถ้าเคยจากเพศใด

sx_rapeX
  - ○ 1. ผู้ชาย
  - ○ 2. ผู้หญิง

27. ท่านคิดว่าตัวท่าน<u>ต้องการ</u>มีเพศสัมพันธ์กับเพศใด

sx_with
- ○ 1.ต้องการมีเพศสัมพันธ์กับ <u>**เพศหญิง**</u>เท่านั้น
- ○ 2.ต้องการมีเพศสัมพันธ์กับ <u>**เพศชาย**</u>เท่านั้น
- ○ 3.ต้องการมีเพศสัมพันธ์กับทั้ง <u>**เพศหญิงและเพศชาย**</u>

28. ท่านทราบหรือไม่ว่าสามารถ<u>**รับแจกถุงยางอนามัยฟรี**</u>ได้จากที่ใด

con_fplc
- ○ 1.ไม่ทราบ
- ○ 2.ทราบ <u>**กรณีทราบ**</u> ท่านเคยได้รับ<u>**การแจกถุงยางอนามัยฟรี**</u>จากที่ใดบ้าง
  - con_fplc1 ○ 1. สถานีอนามัย
  - con_fplc2 ○ 2. โรงพยาบาล
  - con_fplc3 ○ 3. อื่น ๆ ระบุ.................. con_fplc3X ....

29. ท่านทราบหรือไม่ว่าสามารถ<u>**ซื้อ**</u>ถุงยางอนามัยได้จากที่ใด

con_bplc
- ○ 1.ไม่ทราบ
- ○ 2.ทราบ <u>**กรณีทราบ**</u> ท่านเคยซื้อจากที่ใดบ้าง
  - con_bplc1 ○ 1. ร้านค้า / ร้านสะดวกซื้อ (เช่น เซเว่น อีเลฟเว่น)
  - con_bplc2 ○ 2. ร้านขายยา
  - con_bplc3 ○ 3. ตู้จำหน่ายถุงยางอนามัยอัตโนมัติ
  - con_bplc4 ○ 4. อื่น ๆ ระบุ.................. con_bplc4X ....

30. คำถามความรู้และความตระหนักเกี่ยวกับโรคเอดส์

คำถาม	1. ใช่	2. ไม่ใช่
1. การใช้ถุงยางอนามัยสามารถป้องกันการติดเชื้อเอดส์ได้ ใช่หรือไม่	Knlg1 ○	○
2. การมีคู่นอนเพียงคนเดียวที่ไม่มีเชื้อเอดส์เป็นวิธีหนึ่งที่สามารถป้องกันการติดเชื้อเอดส์ได้ ใช่หรือไม่	Knlg2 ○	○
3. ยุงสามารถเป็นพาหะนำเชื้อเอดส์มาสู่คนได้ ใช่หรือไม่	Knlg3 ○	○
4. การกินอาหารร่วมกับผู้ติดเชื้อเอดส์สามารถติดเชื้อเอดส์ได้ ใช่หรือไม่	Knlg4 ○	○
5. คนที่เรามองเห็นว่ามีสุขภาพร่างกายแข็งแรงดีอาจจะเป็นคนที่มีเชื้อเอดส์ได้ใช่หรือไม่	Knlg5 ○	○
6. ในปัจจุบันมียาที่สามารถยับยั้งเชื้อเอดส์ (ยาต้านไวรัส) ได้ ใช่หรือไม่	Knlg6 ○	○
7. การใช้เข็มฉีดยาเสพติดร่วมกัน ทำให้ติดเชื้อเอดส์ได้ ใช่หรือไม่	Knlg7 ○	○

" ขอขอบคุณท่านที่สละเวลาอันมีค่าในการตอบแบบสอบถามมา ณ โอกาสนี้ "
