# Supplementary material for: Prevalence of HIV infection and related risk factors among young Thai men between 2010 and 2011
Source: PLoS One. 2020 Aug 14;15(8):e0237649. doi: 10.1371/journal.pone.0237649 (PMC7428352; doi:10.1371/journal.pone.0237649)
Supplement: S3 File — (PDF) [file pone.0237649.s003.pdf]

Phramongkutklao College of Medicine, Bangkok, Thailand

Data set name: HIV among young Thai men 2010-2011

Variable Information		
**Variables**	**Position**	**Label**
DataGroup	1	*Data Group
Valid	2	*Valid Cases
HIVStatus	3	*00. HIV Status
MSM_Rev	4	33. *MSM Revised
Element	5	*01.0. Type of Element
Age.Cont	6	*02.0. .Age-Cont
LivingStat	7	*4.0.m.LivStat
Region	8	*05.1.0. Region_Full
DistType	9	*05.4. Type of District
Occupation	10	*06.0. Occupation - Full
CivilStatFull	11	*07.0. Marital Status
EducFull_08	12	*08.0.m.Educ-Full
IVDU.Hx	13	*09.0.m.Hx of IVDU
IVDU.Age	14	09.2.Age of first use IVDU
nIVDU.Hx	15	*11.0. nIVDU-Hx
Incar	16	*12.0 Incarceration
HIV_Testing	17	*13.0. Hx of HIV Testing
Transfusion	18	*14.0 Transfusion
Circumcised	19	*15.0. Circumcision
FSX	20	*16.0. FSX.Hx
SxFst_16.2	21	*16.2 FSX - Age Cont
FstSxwho_17	22	*17.1.Who is your first sex
FSW	23	*18.0.m. FSW
SxTotal_19.0	24	*19.0. LifetimeTotal Sex
SxFem_19.2	25	19.1. LifetimeFemale
SxMale_19.3	26	19.2. LifetimeMale
LifeSexAct	27	19.4.0. SxActLifetime
SXPRACT_RECAL	28	20.0. Sexual Activity (12-Mos)
STI.Hx	29	24.0.m.Hx of STI
SxGiftHx	30	25.0.m.Male Sex in exchange for gifts
Coercion	31	26.0.m.Coercion Hx
Sx_Pref	32	27.0.m.Sexual Preference

Phramongkutklao College of Medicine, Bangkok, Thailand

Variable Values		
Value		Label
DataGroup	1	10-Nov
	2	11-May
Valid	0	Invalid
	1	Valid
HIVStatus	0	Negative
	1	Positive
	2a	Ind
MSM_Rev	0	No
	1	Yes
	2a	No Sex
Element	1	Volunteer
	2	Drafted
LivingStat	1	W/ PARENTS
	2	W/ WIFE
	3	W/ RELATIVES
	4	W/ FRIEND
	5	ALONE
	6a	OTHERS
	20	W/ WIFE
Region	1	UPPER NORTH
	1.1	LOWER NORTH
	2	NORTHEAST
	3	EAST
	4	CENTRAL
	5	WEST
	6	SOUTH
	7	BANGKOK
	9.0a	ABROAD
	40	CENTRAL
	60	SOUTH
DistType	1	Inner
	2	Outer
	3	Bangkok
Occupation	1	Student
	2	Worker - Factory
	3	Laborer
	4	Unemployed
	5	Sales
	6	Fisherman/Farmer
	7	Merchant
	8a	Others

	9	technician
	10	freelancer
	11	government employee
	12	contractor
	13	???
	14	school teacher
	15	taxi driver
	16	rubber plantation
	17	HRM
	18	Musician
	60	Fisherman/Farmer
CivilStatFull	1	MARRIED
	2	DIVORCED
	3	WIDOW
	4	SINGLE
	10	MARRIED
EducFull_08	1	NONE
	2	PR 1-6
	3	MA 1-3
	4	MA 4-6
	5	SVoc
	6	DVoc/HVoc
	8	BS
	9	OTHERS
	10	Non-Formal Education
	30	MA 1-3
	50	SVoc
	60	DVoc/HVoc
IVDU.Hx	1	NO
	2	YES
nIVDU.Hx	0	NO
	1	YES
Incar	1	Yes
	2	No
HIV_Testing	1	YES
	2	NO
Transfusion	1	YES
	2	NO
Circumcised	1	YES
	2	NO
FSX	1	YES
	2	NO
FstSxwho_17	1	Friend

	2	wife/lover
	3	FSW
	4	Casual - female
	5	Man-general
	6	man-Commerce
	7	Ladyboy
	8	Others
	20	wife/lover
FSW	1	YES
	2	NO
	10	YES
LifeSexAct	1	Excl Hetero
	2	Excl Homo
	3	Bisex
	4a	No Sex
SXPRACT_RECAL	1	Heterosexual
	2	Homosexual
	3	Bisexual
	777a	No Sex
STI.Hx	1	No
	2	Yes
	10	No
SxGiftHx	1	YES
	2	NO
Coercion	1	YES
	2	NO
Sx_Pref	1	FEMALE
	2	MALE
	3	BOTH
	10	FEMALE
