# Supplementary material for: Prevalence of HIV infection and related risk factors among young Thai men between 2010 and 2011
Source: PLoS One. 2020 Aug 14;15(8):e0237649. doi: 10.1371/journal.pone.0237649 (PMC7428352; doi:10.1371/journal.pone.0237649)
Supplement: Attachment — Submitted filename: Response_Reviwer.docx [file pone.0237649.s005.docx]

**PONE-D-20-03591R1**

**Prevalence of HIV infection and related risk factors among young Thai men between 2010 and 2011**

**PLOS ONE**

Julius Eleazar dC. Jose, Boonsub Sakboonyarat, Khunakorn Kana, Thippawan Chuenchitra, Akachai Sunantarod, Supanee Meesiri, Mathirut Mungthin, Kenrad E. Nelson, Ram Rangsin

**Reviewer #2:**

1. Thank you for your response - and changes. Explanations are clear. Data looks solid, epi claims are now revised. Have not changed to "HIV-infected" throughout, just in select places.
Also, "human immunodeficiency virus" is still in caps in some places. should not be.

**►Response: “**We have rewritten the words “human immunodeficiency virus”[Page 3, Line 2]and “HIV-infected ”[Page 4, Line 63 & Page12, Line 197-198]

**Sincerely Yours,**

**Ram Rangsin, M.D., M.P.H., Dr.P.H.**
